# Improvement of limit of detection in primer extension-based multiplexed mutation assay using capillary electrophoresis

**DOI:** 10.1007/s44211-024-00508-8

**Published:** 2024-02-06

**Authors:** Takahiro Ando, Takahide Yokoi, Chihiro Manri, Takashi Anazawa, Takeshi Ishida

**Affiliations:** Research & Development Group, Hitachi, Ltd., 1-280 Higashi-Koigakubo, Kokubunji-shi, Tokyo 185-8601 Japan

**Keywords:** Capillary electrophoresis, Gene mutation detection, Single nucleotide extension, Cancer diagnosis

## Abstract

One of the challenges in liquid biopsy for early cancer detection is ascribed to the fact that mutation DNA often represents an extremely small ratio of less than 1% compared to wild-type genes in blood. However, in conventional fragment analysis with capillary electrophoresis (CE), the detectable allele frequency could be about 5%. In this work, we developed an original reagent-based fragment analysis with single base extension (SBE) reactions for cancer-associated mutation assay using a commercially available CE device, and investigated on a possibility of improvement of limit of detection (LOD) for genetic mutation. First, after adjustment of reagent conditions for the SBE reactions, the linear relationship between gene template concentration and fluorescence intensity was obtained from 1 to 100 fmol of target genes. Next, from the results of an experiment to detect mutation *EGFR* L858R at abundance ratios of mutant type to wild type (100-fmol template) of 0, 1, 5, and 10%, it was shown that the target gene can be detected with LOD of 0.33%. This high sensitivity was realized in part by separating fluorescently labeled substrates into an individual tube for an each-colored SBE reaction. Moreover, mutations *EGFR* L858R and *KRAS* G12V were simultaneously detected at sensitivities equivalent to LODs of 0.57 and 0.47%, respectively. These results indicate that < 1% of mutations in multiplex gene mutations can be simultaneously detected, and that possibility suggests that the developed method can be used in clinical practice for detecting cancers.

**Graphical abstract:**

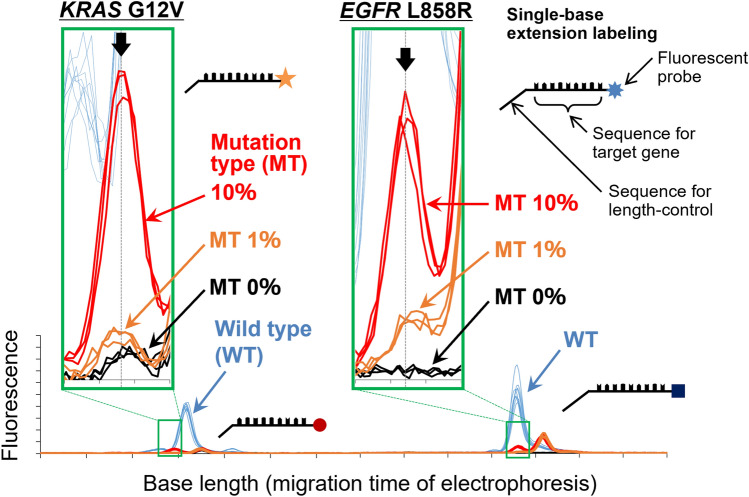

## Introduction

Cancer has a significant impact on public health worldwide. Early detection and intervention are likely to be the most effective means for reducing mortality due to cancer. However, development of methods for non-invasive detection of early stage tumors has remained a challenge. Currently, liquid biopsy with circulating tumor DNA (ctDNA) profiling is considered to hold great promise in revolutionizing clinical oncology [1–3]. Cell-free DNA (cfDNA) is released into the blood circulation by both physiological and pathological mechanisms, and in cancer patients, a fraction of blood-borne cfDNA is tumor-derived and called ctDNA.

One of the challenges concerning liquid biopsy is the fact that ctDNA often represents an extremely small ratio (< 1%) compared to normal circulating cfDNA in blood [4, 5]. Therefore, a highly sensitive assay is required to quantify a rare mutated gene (mutation type, MT) in a large excess of normal genes (wild type, WT). For example, digital PCR (dPCR) is a sensitive and robust method that enables the detection and quantitation of targeted DNA mutations in a variety of clinical samples. Also, next generation sequencing (NGS) has its benefits in terms of massively and comprehensively detecting different types of mutations at once, while its sensitivity is generally lower than dPCR. In the meantime, capillary electrophoresis (CE) is still a traditional method for more straightforward and robust assay for detecting genetic mutation, although the sensitivity in the case of quantitative detection of rare mutations is low.

Previous studies have revealed that Sanger sequencing by CE has sensitivity with the limit of detection (LOD) for gene mutation of 5–20% MT/WT [6–10]. This low sensitivity is mainly due to overlapping adjacent fluorescent signals in an electropherogram, by which one of the signals forms a high background signal for the other. Even in the case of fragment analysis by CE, such as the SNaPshot multiplex mutation-detection system, where adjacent peaks are sufficiently separated in an electropherogram, LOD is limited to about 5% MT/WT [11, 12]. As for high-sensitivity sequencing detection, for example, a previous study reported that a real-time PCR-based allele enrichment technology was effective in enriching rare variant sequences with LOD as low as 0.1% MT/WT. However, the applicable mutations and the multiplexing capabilities are limited [13–15]. Thus, CE methodology has been generally used for qualitative evaluation of genetic mutations, and so far accurate detection with high sensitivity has not been achieved.

Molecular diagnosis with CE has a great potential for clinical use because it has short turn-around-time (TAT) and is relatively inexpensive, which are both essential characteristics for practical use [10]. Therefore, in the present study, we developed an original reagent-based fragment analysis with single base extension (SBE) reactions for various mutation assays, and we investigated the possibility of improving LOD with this analysis method.

## Experimental

### Amplification of DNA template

OncoSpan DNA Reference Standard (HD827, Horizon), which contains genetic mutations associated with cancer, was used as a standard sample. TaKaRa Ex Taq PCR kit was used to amplify, the target sequences of mutations *EGFR* L858 and *KRAS* G12. PCR primer pairs (5’-GCAGCATGTCAAGATCACAGATT-3′ and 5′-CCTCCTTCTGCATGGTATTCTTTCT-3′) and (5′-AGGCCTGCTGAAAATGACTGAATAT-3′ and 5′-GCTGTATCGTCAAGGCACTCTT-3′) were used for *EGFR* L858 and *KRAS* G12, respectively. Each PCR reaction contained TaKaRa Ex Taq (5 U/μL), 4-mM dNTP mixture, 1 × Ex Taq buffer, 1.25-μL standard sample template, and 0.3-μM primers in 50-μL final volume. Thermal cycling was performed with a SimpliAmp thermal cycler (Thermo Fisher Scientific) under the following conditions: 94 °C for 5 min, 25 × (94 °C for 10 s, 55 °C for 30 s, 72 °C for 60 s), and 72 °C for 7 min. The amplicon lengths were designed as 78 and 80 bps. In the amplification of mutation *EGFR* L858, the PCR products were transformed into *E. coli* using a TOPO TA Cloning Kit (Thermo Fisher Scientific), and the plasmid DNA was extracted using a QIAprep miniprep kit (Qiagen). For the mutated genes, *EGFR* L858Q and L858P, which do not include in OncoSpan DNA Reference Standard, a site directed mutagenesis was applied to the obtained plasmid using a PrimeSTAR Mutagenesis Basal Kit (Takara). Thus, using these plasmids for DNA templates, the target base sequence was amplified by PCR under the above-mentioned cycling conditions. The PCR products, i.e., DNA template including target sequence, were purified using NucleoSpin Gel and PCR Clean-up (Takara Bio). The length and concentration of the PCR products were then measured using a TapeStation (Agilent). In case extra bands in the PCR-amplified products were observed, the entire amount of the products was subjected to electrophoresis on an agarose gel, the PCR products with the target base length were cut out with a razor blade, and the length and concentration of the products were measured again. Quantitative PCR was used for copy-number measurement, with a QuantStudio instrument (Thermo Fisher Scientific), under the following conditions: 95 °C for 30 s, followed by 40 × (95 °C for 5 s, and 60 °C for 34 s).

### SBE reaction

SBE reaction is performed using a primer that bind up to the genetic site of interest. The chemistry results in the extension of each SBE primer by one base, using fluorescently labeled dideoxyribonucleoside triphosphate (ddNTP), to reveal the identity of the nucleotide base on the template DNA. The final concentrations of the constituents in the SBE-reaction mixture (after adjustment of concentration of ddNTP reagents) are listed in Table 1. Since a fluorescent signal depends on the amount of fluorescent probe, we used non-labeled (*i.e.,* not fluorescent) ddNTPs to adjust the fluorescence intensity, so that fluorescence intensity changes linearly with gene-template concentration. To activate SBE reactions, the reaction mixture was subjected to thermal cycling with a SimpliAmp thermal cycler (Thermo Fisher Scientific) under the following conditions: 25 × (96 °C for 10 s, 50 °C for 5 s, 60 °C for 30 s). For the SBE reactions, Therminator (New England Biolabs) was used as a polymerase for incorporating fluorescent-labeled substrates [16]. To avoid any signal noise due to misincorporation of ddNTPs or misestimation by color-conversion error, fluorescently labeled ddNTPs (R6G-ddATP, ROX-ddUTP, R110-ddGTP, and TAMRA-ddCTP) were separated into an individual tube for the SBE reaction of each color. The reason for the use of ddUTP instead of ddTTP were easy market availability obtained from the same manufacture as the other three ddNTPs and the substitutability [17], although it requires careful adjustment of the concentration before measurement. The primer sequences used for the SBE reactions are shown in Tables 2 and 3. All the SBE primers were purchased from Sigma–Aldrich in PAGE purification grade, except EGFR L858 Fw (90) primer from Integrated DNA Technologies (IDT) in HPLC purification grade.

**Table 1:** Reagents used for SBE reactions. Concentrations of ddNTP reagents were adjusted to obtain linear relationship between gene-template concentration and fluorescence intensity

Materials		Final concentration
DNA polymerase	• Therminator DNA polymerase • 10 × Therminator buffer (#M0261L, New England Biolabs)	1 U
ddNTP	• R6G-ddATP • ROX-ddUTP • R110-ddGTP • TAMRA-ddCTP Fluorescence-labeled nucleotide (Perkin Elmer)	0.1 μM 4 μM 0.1 μM 0.1 μM (unless otherwise described)
	• Non-labeled ddATP • Non-labeled ddGTP Dideoxynucleoside triphosphate set (#3732738001, Merck)	1 μM 10 μM
Primer	(Described in Tables 2 and 3)	0.2 μM (unless otherwise described)
DNA template (PCR product)	(Described in “Amplification of DNA template”)	0 fmol 0.1 fmol 0.3 fmol 1 fmol 3 fmol 5 fmol 10 fmol 30 fmol 100 fmol (= 100%)
D.W	#10977-015 (Invitrogen)

**Table 2:** Primer sequence used in SBE experiments

Gene	Primer name	Sequence (5′ → 3′) Italic and underlined: target genetic sequence Black: SP6 promoter (24 mer) Bold: additional base for size adjustment	Primer size (bp)	Targeted base for detection	
				WT	MT
*EGFR* L858R (c.2573 T > G)	EGFR L858—Fw1	CAAGCTATTTAGGTGACACTATAG*CAGCATGTCAAGATCACAGATTTTGGGC*	52	T	A, C, G
	EGFR L858—Fw2	**CAGGAAACAGCTATGAC**CAAGCTATTTAGGTGACACTATAG*GCATGTCAAGATCACAGATTTTGGGC*	67	T	G
*KRAS* G12V (c.35G > T)	KRAS G12—Fw	CAAGCTATTTAGGTGACACTATAG*GAATATAAACTTGTGGTAGTTGGAGCTG*	52	G	T

**Table 3:** Primer sequences used for SBE reactions

#	Gene	Primer name	Sequence (5′ → 3′) Italic and underlined: target genetic sequence Black: SP6 and linker promoter Bold: additional base for size adjustment	Primer size (bp)	Targeted base for detection	
					WT	MT
1	*EGFR* L858R (c.2573 T > G)	EGFR L858 Fw (50)	ATGGGTGGACGTGACACTATAG*CAGCATGTCAAGATCACAGATTTTGGGC*	50	T	G
2		EGFR L858 Fw (55)	ATGGGTGGACTTTAGGTGACACTATAG*CAGCATGTCAAGATCACAGATTTTGGGC*	55		
3		EGFR L858 Fw (60)	ATGGGTGGACAGCTATTTAGGTGACACTATAG*CAGCATGTCAAGATCACAGATTTTGGGC*	60		
4		EGFR L858 Fw (65)	ATGGGTGGAC**AGT**CAAGCTATTTAGGTGACACTATAG*CAGCATGTCAAGATCACAGATTTTGGGC*	65		
5		EGFR L858 Fw (70)	ATGGGTGGAC**CGGCCAGT**CAAGCTATTTAGGTGACACTATAG*CAGCATGTCAAGATCACAGATTTTGGGC*	70		
6		EGFR L858 Fw (75)	ATGGGTGGAC**AACGACGGCCAGT**CAAGCTATTTAGGTGACACTATAG*CAGCATGTCAAGATCACAGATTTTGGGC*	75		
7		EGFR L858 Fw (80)	ATGGGTGGAC**CGTAAAACGACGGCCAGT**CAAGCTATTTAGGTGACACTATAG*CAGCATGTCAAGATCACAGATTTTGGGC*	80		
8		EGFR L858 Fw (85)	ATGGGTGGAC**TATGACGTAAAACGACGGCCAGT**CAAGCTATTTAGGTGACACTATAG*CAGCATGTCAAGATCACAGATTTTGGGC*	85		
9		EGFR L858 Fw (90)	ATGGGTGGAC**ACAGCTATGACGTAAAACGACGGCCAGT**CAAGCTATTTAGGTGACACTATAG*CAGCATGTCAAGATCACAGATTTTGGGC*	90		
10		EGFR L858 Fw (95)	ATGGGTGGA**CAGGAAACAGCTATGACGTAAAACGACGGCCAGT**CAAGCTATTTAGGTGACACTATAG*CAGCATGTCAAGATCACAGATTTTGGGC*	95		
11		EGFR L858 Fw (100)	ATGGGTGGA**CTCTCCAGGAAACAGCTATGACGTAAAACGACGGCCAGT**CAAGCTATTTAGGTGACACTATAG*CAGCATGTCAAGATCACAGATTTTGGGC*	100		

### Fragment analysis with CE sequencer

**Fig. 1:**
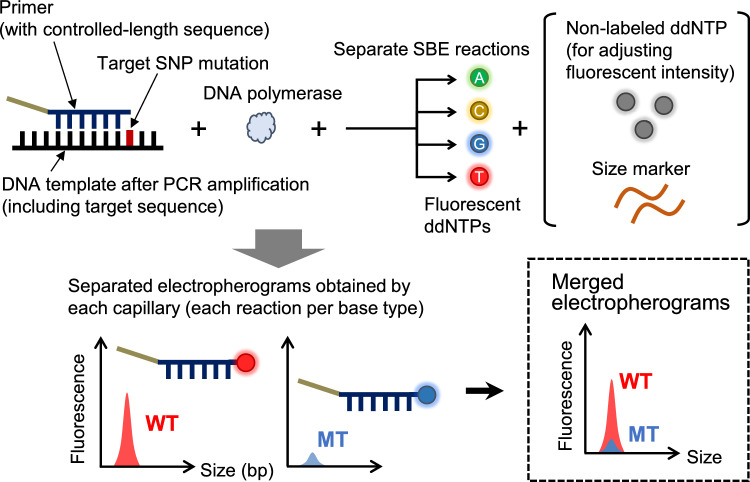
Workflow of genetic-mutation detection using a CE sequencer

Excess primers and unincorporated dNTPs were inactivated with shrimp alkaline phosphatase (SAP). The reaction product (10 µL) was treated with 1 µL of SAP (#2660A, Takara Bio) and incubated at 37 °C for 1 h and then 75 °C for 15 min. To the SAP-treated samples (0.5 µL), a mixture of each 0.5 µL of two kinds of size standard (#4324287, Thermo Fisher Scientific and #DG5001, Promega) and 8.5 µL of Hi-Di Formamide (#4311320, Thermo Fisher Scientific) was added (total volume of 10 µL). Based on preliminary experiments, a solution of the former size standard at fourfold dilution was used. After heat treatment of the mixture at 95 °C for 5 min, the heat-treated solution was subjected to fragment analysis using a compact CE sequencer DS3000 (Hitachi High-Tech). The DS3000 has a hardware configuration of four 36-cm-long capillaries that can simultaneously detect six colors. Polymer 4 was used as the sequencing polymer. The electrophoresis conditions were set according to the results of preliminary studies: injection voltage of 1.6 kV, run voltage of 13 kV, oven temperature of 60 °C, injection time of 9 s, run time of 1,930 s, and delay time of 1 s. The raw data (msd file) of the obtained electropherogram were converted to CSV files using our original software. The workflow of genetic-mutation detection using the CE sequencer and signal analysis is shown in Fig. 1.

Regarding the fluorescent labels used in the SBE reactions, the spectra of fluorescence emitted from different fluorophores are not completely separated from each other, and some overlap in a manner known as “spectrum crosstalk” [18]. To cancel the spectrum crosstalk, fluorescently labeled ddNTPs (R6G-ddATP, ROX-ddUTP, R110-ddGTP, and TAMRA-ddCTP) and two kinds of size standards were independently electrophoresed to obtain six types of fluorescence-spectrum data, which was subjected to inverse-matrix calculation for color conversion. Based on data matrix ${\varvec{A}}$ for N bins of fluorescent wavelength bands × six types of obtained fluorescent labels, each data matrix ${\varvec{Y}}$ (N × 1 matrix) was multiplied by inverse matrix ${{\varvec{A}}}^{-}$ from the left side for color-conversion processing. The determinant is given as:

$${\varvec{A}}=\left(\begin{array}{cc}\begin{array}{cc}{a}_{1, 1}& {a}_{\mathrm{1,2}}\\ {a}_{\mathrm{2,1}}& {a}_{\mathrm{2,2}}\end{array}& \begin{array}{cc}\cdots & {a}_{\mathrm{1,6}}\\ \cdots & {a}_{\mathrm{2,6}}\end{array}\\ \begin{array}{cc}\vdots & \vdots \\ {a}_{{\text{N}}, 1}& {a}_{{\text{N}},2}\end{array}& \begin{array}{cc}\ddots & \vdots \\ \cdots & {a}_{{\text{N}}, 6}\end{array}\end{array}\right), {\varvec{X}}=\left(\begin{array}{c}{\text{I}}\left(F1\right)\\ {\text{I}}\left(F2\right)\\ \begin{array}{c}\vdots \\ {\text{I}}\left(F6\right)\end{array}\end{array}\right),{\varvec{Y}}=\left(\begin{array}{c}{\text{I}}\left(C1\right)\\ {\text{I}}\left(C2\right)\\ \begin{array}{c}\vdots \\ {\text{I}}\left(C{\text{N}}\right)\end{array}\end{array}\right).$$

From ${\varvec{Y}}={\varvec{A}}\times {\varvec{X}}$, it is possible to obtain ${\varvec{X}}={{\varvec{A}}}^{-}\times {\varvec{Y}}$. The number of equations (for N bins of fluorescence wavelength bands) is greater than the number of unknowns (six fluorescent labels), resulting in multiple solutions for matrix ${\varvec{X}}$. Therefore, a unique solution that minimizes the square error of all equations, that is, generalized-inverse matrix ${{\varvec{A}}}^{-}$ can be obtained by the following equation:

$${{\varvec{A}}}^{-}={\left({{\varvec{A}}}^{{\varvec{T}}}\times {\varvec{A}}\right)}^{-1}\times {{\varvec{A}}}^{{\varvec{T}}}$$

where matrix ${{\varvec{A}}}^{{\varvec{T}}}$ is the transposed matrix of ${\varvec{A}}$. Thus, it is possible to calculate generalized-inverse matrix ${{\varvec{A}}}^{-}$ from data matrix ${\varvec{A}}$ obtained by independently electrophoresing the six types of fluorescent labels. The color conversion calculation is performed using generalized-inverse matrix ${{\varvec{A}}}^{-}$ to obtain a unique solution for ${\varvec{X}}$.

## Results and discussion

**Fig. 2:**
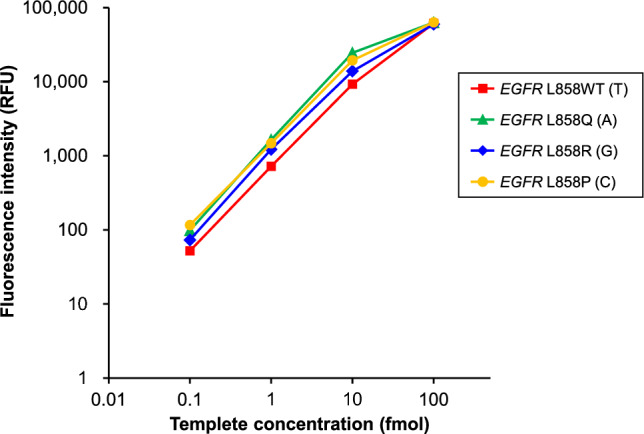
Relationship between DNA-template concentration and peak-fluorescence intensity after experimental adjustment of ddNTP reagent

At first, we adjusted the reagent conditions for SBE reactions using *EGFR* L858 templates that assumed a quantitative detection of wild type (WT) and mutant type (MT). This adjustment is to establish a state in which fluorescence intensity changes linearly with template concentration, so that the quantitative ratio of MT/WT can be obtained. Using *EGFR* L858 primer (named EGFR L858—FW1), wild-type *EGFR* L858WT was tagged T and three mutants of *EGFR* L858Q, *EGFR* L858R and *EGFR* L858P were labeled A, G, and C, respectively. Peak values of fluorescence signals at PCR-amplified template concentrations of 0.1, 1, 10, and 100 fmol were measured by independent capillary electrophoresis. Figure 2 shows a relationship between DNA template-concentration and peak fluorescence intensity after experimental adjustment of ddNTP reagent. These plots show rough signal linearity from 0.1 to 100 fmol. At template concentration of 100 fmol, some of the fluorescence intensities might have been saturated. This issue is expected to be resolved by expanding the dynamic range of CE sequencer, and that expansion is currently under investigation [19]. In the following experiments, we thus decided to use the above reagent conditions.

**Fig. 3:**
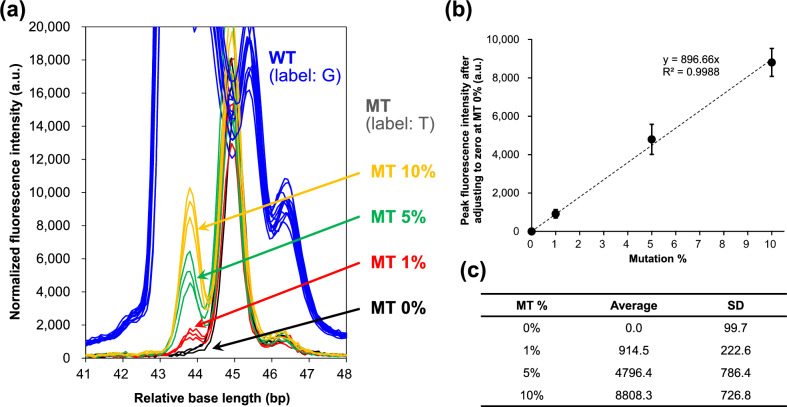
Results of experimental detection of mutation *EGFR* L858R (*n* = 3): **a** electropherograms, **b** linearity of calibration curve and **c** relation between MT % and fluorescence intensity

Figure 3 shows results of an experiment to detect mutation *EGFR* L858R at abundance ratio of MT/WT of 0, 1, 5, and 10%, where a concentration of WT template was 100 fmol. The concentration of primer EGFR L858 Fw (50) used in the SBE reaction was 0.4 µM in this experiment. The inverse-matrix calculation of the color conversion was applied to an electropherogram and Promega size standard was used for base length sizing. A peak-fluorescence intensity of the sizing standard at 100 bp was used for normalizing the signals. As shown in the electropherogram in Fig. 3a, fluorescence signal intensity increased as quantitative ratio of MT/WT was increased from 0 to 1%, 5, and 10%. A relatively large fluorescent signal observed around base length of 45 bp exists regardless of the MT percentage, and it would be due to the unevenness of the primer and/or the low purity of the ddNTP substrate leading to a potential two-base extension. Detailed examination and adjustment of reagents will be necessary in the future to avoid the fluorescent signal, but it is not the subject of analysis here because the signals were not changed depending on the proportion of mutation and no effects on the analysis. Figures 3b, c show the results of a calibration curve and its standard deviation (SD) values, in which peak fluorescence intensity at MT/WT ratio of 0% was considered as the baseline value. The LOD was defined as three times the SD for 0% mutation (S/N ratio = 3) [19], and the LOD calculated from the approximation line was 0.33%. These results show that the target gene *EGFR* L858R can be detected with significantly higher sensitivity than the conventional sensitivity (5%). This high sensitivity was realized partly by the fact fluorescently labeled substrates were separated into individual tubes for the SBE reactions for each color to avoid any signal noise due to misincorporation of ddNTPs and misestimation by color-conversion error. As for the sizing of base length, a peak was observed at a shorter base length (approximately 43 to 45 bp) than the primer size (52 bp) that should be detected. This observation is presumably because the Promega size standard is marked at 60 bp or more, and the accuracy of calculated sizing would be poor for less than 60 bp. Therefore, the horizontal axis was expressed as relative base length in the Fig. 3a. Since it is easy to correct the size standard for errors in detection timing and base length, and it is not important in terms of detecting *EGFR* L858R mutation, this discrepancy of base lengths is not considered a major issue.

**Fig. 4:**
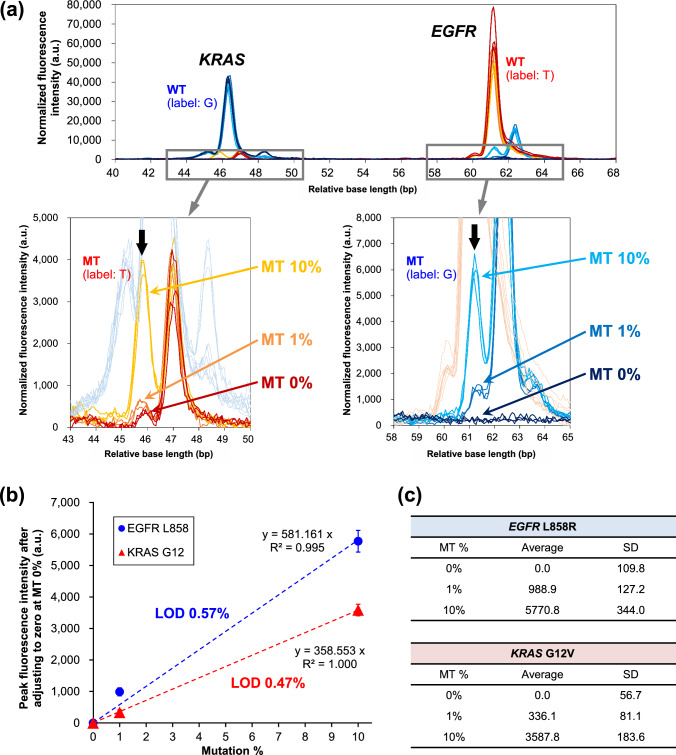
Results of simultaneous experimental detection of mutations *EGFR* L858R and *KRAS* G12V (*n* = 3): **a** electropherograms, **b** linearity of calibration curve, and **c** relation between MT % and fluorescence intensity

To investigate whether multiplexed genetic mutations can be simultaneously detected with an equivalent sensitivity as mentioned above (0.33%), SBE primers separated by 15 bp were used as two mutation targets (2 plex), *EGFR* L858R and *KRAS* G12V, for which the primers were EGFR L858—FW2 and KRAS G12—FW1, respectively. Figure 4 shows results of an experiment to detect mutations *EGFR* L858R and *KRAS* G12V at abundance ratio of MT/WT of 0, 1, and 10%, where a concentration of WT template was 100 fmol. As shown in the electropherogram in Fig. 4a, the fluorescence signals derived from the detection of *EGFR* L858R and *KRAS* G12V are clearly observed at base lengths about 15 bp apart. Also, fluorescence-signal intensity almost linearly increases as MT ratio increases from 0 to 1 and 10%. The fluorescence-signal peaks indicated by black arrows indicate the MT detection of *EGFR* L858R and *KRAS* G12V. Note that the enlarged views in the lower part of Fig. 4a are shown in different colors for WT signals so as to make it easier to see the difference in fluorescence signals. Figure 4b, c show the results of a calibration curve and its SD values. A peak fluorescence intensity at MT/WT ratio of 0% was considered as the baseline value, and thus the difference how much the signals exceed from the baseline was focused on. By calculating the LODs in the same manner described above, the obtained LODs were 0.57 and 0.47% for *EGFR* L858R and *KRAS* G12V, respectively. The reliability of linearity should be carefully assessed in further studies. This is because the present calibration curve was composed of only two valid plots, and then there would be a necessity of investigating low-concentration mutations (< 1%) for ensuring precise calibration. Nevertheless, these results surely show a potential that < 1% of mutations in multiplex gene mutations can be simultaneously detected in the present methodology. Since the length of SBE primers can be arbitrarily controlled, the number of detectable genetic mutations can, in principle, be increased to all the length range of bases that can be used in fragment analysis [20–22]. Thus, the improved detection sensitivity of this method is expected to be sufficient for diagnosis of various tumors.

**Fig. 5:**
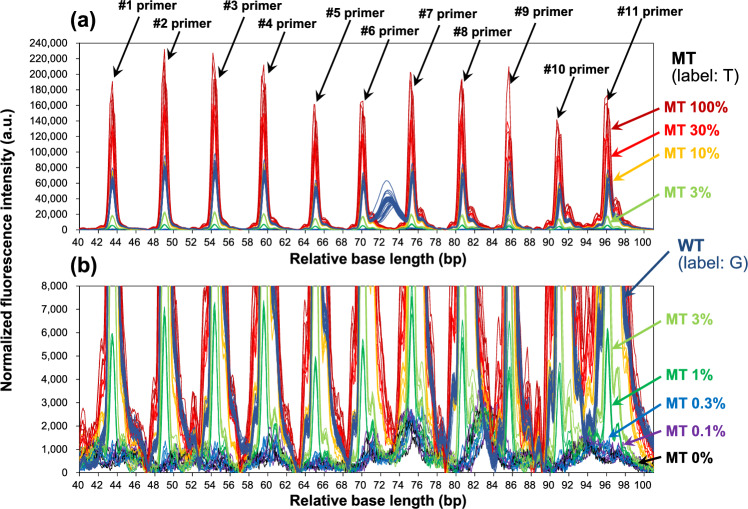
Overlaid electropherograms of experimental detection of mutation *EGFR* L858 using 11-length primers (*n* = 4): **a** overall and **b** enlarged electropherograms

Aiming to further improve LOD of SBE-based mutation detection, we also investigated whether a specific target gene, *EGFR* L858R, could be detected using 11 primers prepared in increments of 5 bp (as shown in Table 3). Figure 5 shows the electrophoresis results for MT ratios of 0, 0.1, 0.3, 1, 3, 10, 30, and 100%. According to the results of the preliminary experiment, the concentration of primer used in the SBE reactions was set to 0.4 µM, and the concentration of R110-ddGTP, which labels base G, was 1 µM. As shown in the overall image of the electropherogram in Fig. 5a, fluorescence signals were detected for each of the 11 primers prepared at 5-bp intervals. The fluorescence signal appearing around the base length of 73 bp is considered to be noise due to dye blobs [23, 24] derived from unremoved fluorescent labels. From the magnified view of the electropherogram in Fig. 5b, it is clear that all the primers show fluorescence signals at MT/WT of 1%. Also, there is some difference in the intensity of the signal detected at a position near the target from that in Fig. 3a around base length of 45 bp. Although a more detailed investigation is necessary, this would be due to the difference of concentrations of primer and ddNTP substrate. As described above, the LOD calculated from each primer’s calibration curve using primers #1 to #11 is 0.66 ± 0.046%, and it varies from 0.033 to 0.205% for the 11 primers. In other words, although any primer can be detected at sensitivity of < 1%, when a specific type of base-length primer is used, mutations can be detected at LOD of 0.033%, but in some cases it can be as low as 0.205%. This result indicates that mutation detection may be not reliable, suggesting a low accuracy of MT %. On the other hand, as an analysis method to solve this inaccuracy issue, the fluorescence-signal peak values obtained from the 11 primers for one type of target gene (*EGFR* L858R) were summed up. By summing the peak signals, the LOD was calculated to be 0.050 ± 0.001% (*n* = 4), indicating the value is extremely small and its deviation is quite small. Therefore, the proposed method of detecting mutations with multiple primers for one gene panel is thought to be useful as a means of stably detecting mutations in a single electrophoresis.

## Conclusion

In this work, an original reagent-based fragment analysis with SBE reactions for a multiplexed mutation assay using a commercially available CE device was developed, and it was shown experimentally that the LOD for genetic mutation of this analysis method is improved compared to conventional methods. From the results of an experiment to detect mutation *EGFR* L858R (at abundance ratios of MT/WT of 0, 1, 5, and 10%) by separating fluorescently labeled substrates for each color, it was shown that the target gene *EGFR* L858R can be detected at LOD of 0.33%. Additionally, mutations *EGFR* L858R and *KRAS* G12V were simultaneously detected at an equivalent sensitivity levels as LODs of 0.57 and 0.47%, respectively. These results indicate that < 1% of mutations in multiplex gene mutations can be simultaneously detected, and that possibility suggests that the proposed detection method can be used in clinical practice for detecting cancers.

## Data Availability

The data sets generated during and/or analyzed during the current study are available from the corresponding author on reasonable request.
